# Supplementary material for: Structural basis of substrate recognition and translocation by human very long-chain fatty acid transporter ABCD1
Source: Nat Commun. 2022 Jun 8;13:3299. doi: 10.1038/s41467-022-30974-5 (PMC9177597; doi:10.1038/s41467-022-30974-5)
Supplement: Supplementary file 1 — Supplementary Information [file 41467_2022_30974_MOESM1_ESM.pdf]

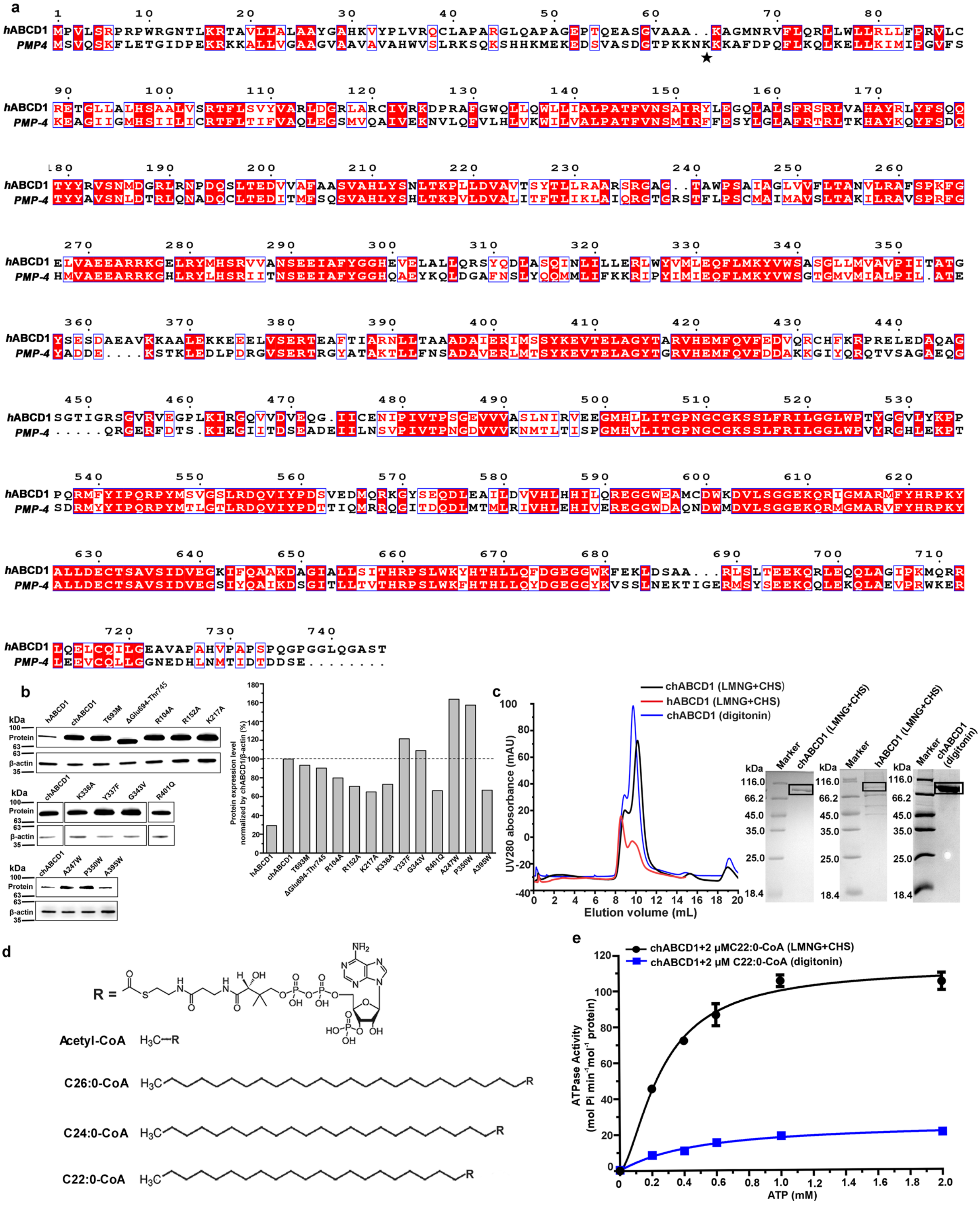

Supplementary Fig. 1 Construction and purification of ABCD1. a, Multiple-sequence alignment of human ABCD1 and *Caenorhabditis elegans* PMP-4. A chimeric version of ABCD1

was constructed by replacing the N-terminal 63 residues with the N-terminal 65 residues of PMP-4 The N-terminal Lys65 of PMP-4 is indicated by an asterisk. **b,** The Western blot analysis to quantify the expression levels of the variants in the total cell extracts, β-actin served as an internal control (n=1). The immunoblot of hABCD1, chABCD1 and mutants of chABCD1 were completed in three different experiments, and controls of β-actin and chABCD1 were run on each blot, and the PVDF membranes were sliced into stripes according to a pre-stained protein marker. The expression levels were shown in the bar chart normalized by chABCD1/β-actin (%). The Western blot analysis results is a representatives (n=1) of many times optimized experiments. Source data are provided as a Source Data file. **c.** Representative size-exclusion chromatography profiles and SDS-PAGE analysis of chimeric ABCD1 (chABCD1) and human ABCD1 (hABCD1) using a Superdex 200 increase 10/300 GL SEC column. The peak fractions around 10 mL for human ABCD1 and 10.2 mL for chimeric ABCD1 were pooled and concentrated for biochemical and structural studies. The purified protein was visualized by Coomassie-blue stained SDS-PAGE and bands for chimeric ABCD1 and human ABCD1 were indicated by black rectangles. The chromatogram and SDS-PAGE are representatives of > 5 independent experiments that showed similar results. **d,** Structural formulas of compounds used in the ATPase activity assays. **e,** Substrate concentration-dependent ATPase activity of chABCD1 upon addition of C22:0-CoA in detergent of digitonin or LMNG+CHS. The data points were fitted with a Michaelis-Menten equation. All data points represent means of three independent measurements (n=3). Error bars indicate standard deviation.

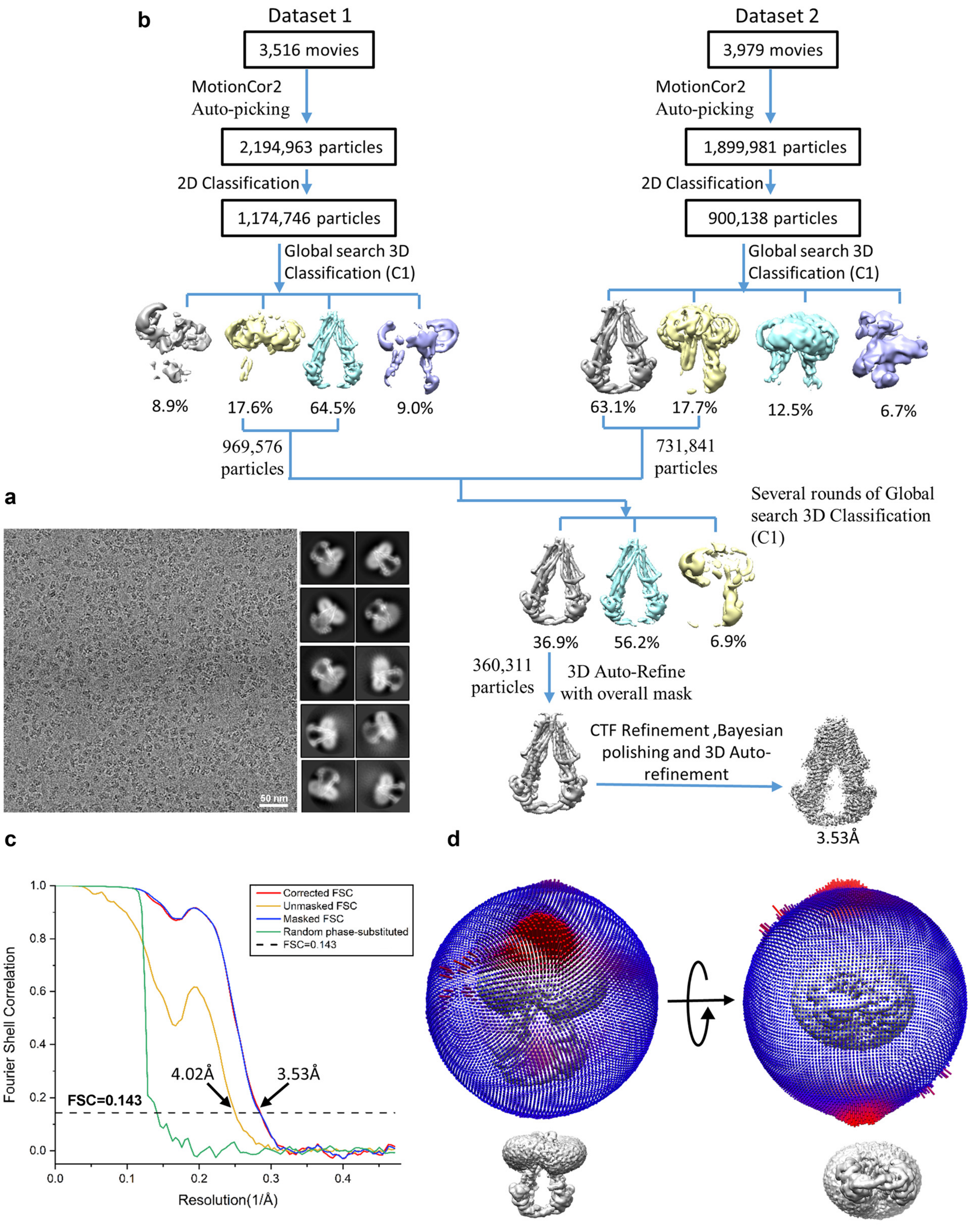
b
Dataset 1
3,516 movies
MotionCor2
Auto-picking
2,194,963 particles
2D Classification
1,174,746 particles
Global search 3D
Classification (C1)
8.9%
17.6%
64.5%
9.0%
969,576
particles
Dataset 2
3,979 movies
MotionCor2
Auto-picking
1,899,981 particles
2D Classification
900,138 particles
Global search 3D
Classification (C1)
63.1%
17.7%
12.5%
6.7%
731,841
particles
Several rounds of Global
search 3D Classification
(C1)
36.9%
56.2%
6.9%
360,311
particles
3D Auto-Refine
with overall mask
CTF Refinement ,Bayesian
polishing and 3D Auto-
refinement
3.53Å
a
50 nm
c
Corrected FSC
Unmasked FSC
Masked FSC
Random phase-substituted
FSC=0.143
Fourier Shell Correlation
Resolution(1/Å)
FSC=0.143
4.02Å
3.53Å
1.0
0.8
0.6
0.4
0.2
0.0
0.0
0.1
0.2
0.3
0.4
d

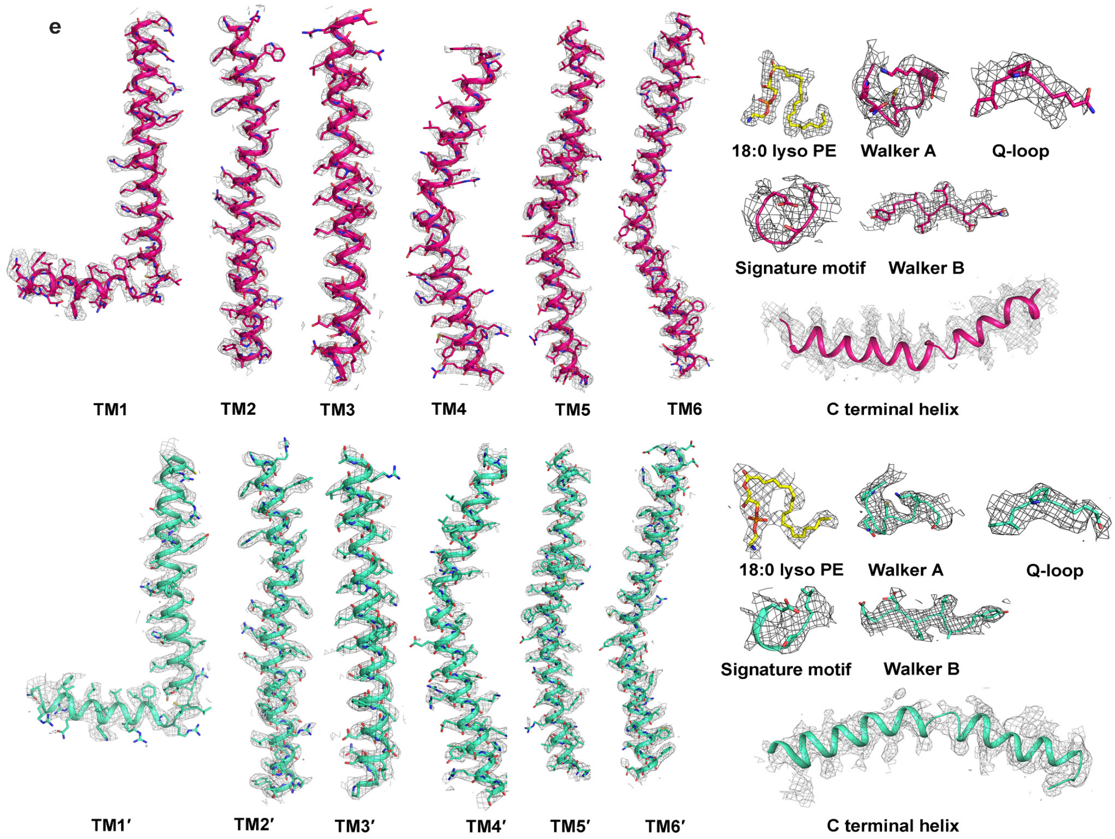

Supplementary Fig. 2 Data processing and model building of apo-form ABCD1. a, Representative cryo-EM micrographs and 2D averages. Bar: 50 nm. The micrograph is a representative of 7,459 cryo-EM images. **b,** Flowchart for cryo-EM data processing. **c,** Gold-standard FSC curve for apo-form ABCD1 map generated using RELION 3.1. **d,** Euler angle distribution of the classified particles used for the final 3D refinement of the overall map. **e,** cryo-EM maps for representative segments of apo-form ABCD1. The structure was reconstructed with C1 symmetry, so all the representative segments in two subunits are thus presented. Contour levels are set at 5σ for TM1-6 and two phosphatidyl ethanolamine (PE) molecules, and at 4σ for the consensus motif in NBDs including Walker A, Q-loop, signature motif, and Walker B. The contour level for C-terminal helix is set at 3σ.

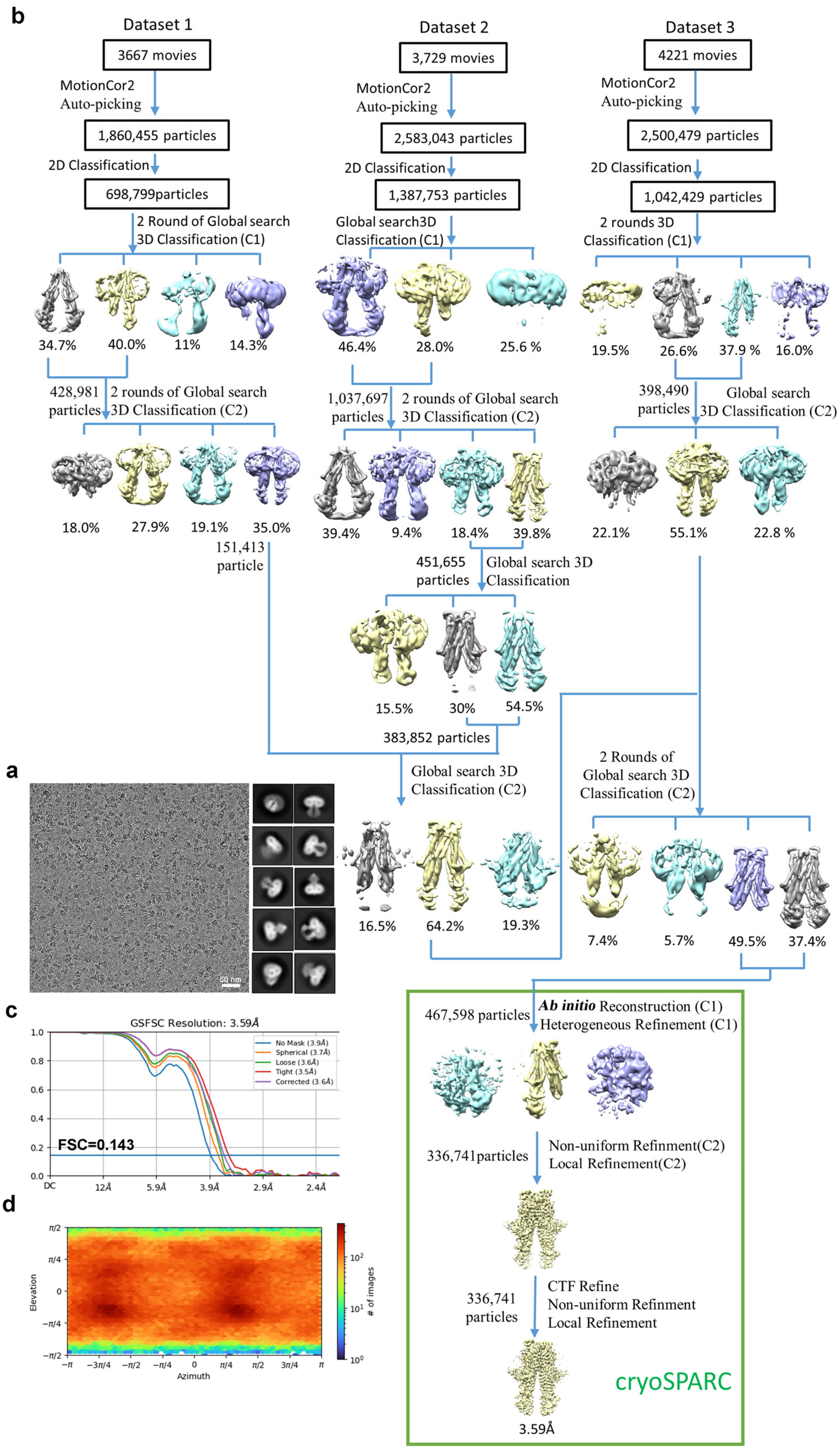
b
Dataset 1
3667 movies
MotionCor2
Auto-picking
1,860,455 particles
2D Classification
698,799particles
2 Round of Global search
3D Classification (C1)
34.7%
40.0%
11%
14.3%
428,981 particles
2 rounds of Global search 3D Classification (C2)
18.0%
27.9%
19.1%
35.0%
151,413 particle
Dataset 2
3,729 movies
MotionCor2
Auto-picking
2,583,043 particles
2D Classification
1,387,753 particles
Global search3D Classification (C1)
46.4%
28.0%
25.6 %
1,037,697 particles
2 rounds of Global search 3D Classification (C2)
39.4%
9.4%
18.4%
39.8%
451,655 particles
Global search 3D Classification
15.5%
30%
54.5%
383,852 particles
Global search 3D Classification (C2)
16.5%
64.2%
19.3%
Dataset 3
4221 movies
MotionCor2
Auto-picking
2,500,479 particles
2D Classification
1,042,429 particles
2 rounds 3D Classification (C1)
19.5%
26.6%
37.9 %
16.0%
398,490 particles
Global search 3D Classification (C2)
22.1%
55.1%
22.8 %
2 Rounds of Global search 3D Classification (C2)
7.4%
5.7%
49.5%
37.4%
467,598 particles
Ab initio Reconstruction (C1)
Heterogeneous Refinement (C1)
336,741particles
Non-uniform Refinment(C2)
Local Refinement(C2)
336,741 particles
CTF Refine
Non-uniform Refinment
Local Refinement
cryoSPARC
3.59Å
a
50 nm
c
GSFSC Resolution: 3.59Å
No Mask (3.9Å)
Spherical (3.7Å)
Loose (3.6Å)
Tight (3.5Å)
Corrected (3.6Å)
FSC=0.143
DC
12Å
5.9Å
3.9Å
2.9Å
2.4Å
d
Elevation
Azimuth
# of images

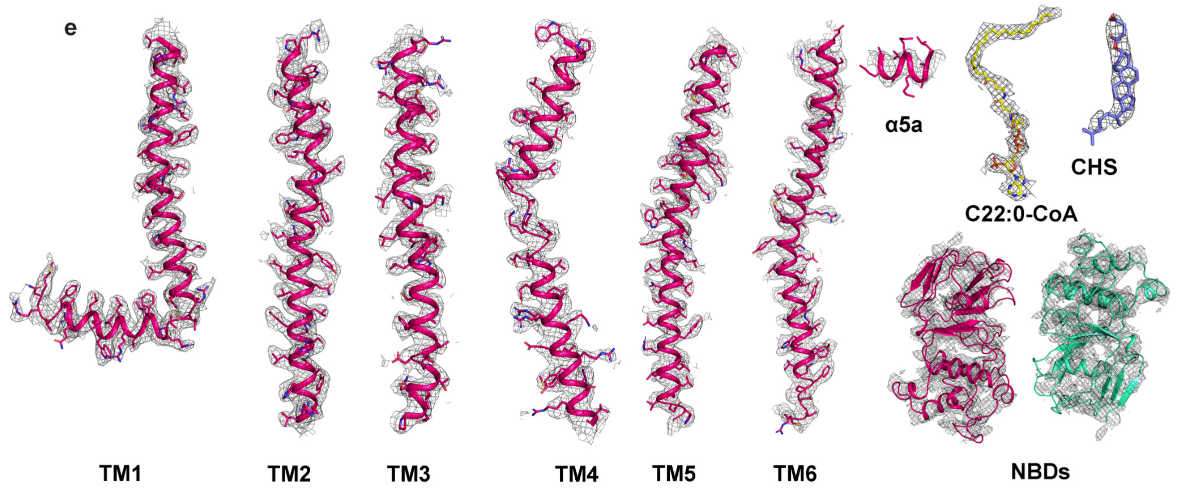

Supplementary Fig. 3 Data processing and model building of C22:0-CoA-bound ABCD1. a, Representative cryo-EM micrographs and 2D averages. Bar: 50 nm. The micrograph is a representative of 11,617 cryo-EM images. **b,** Flowchart for cryo-EM data processing. **c,** Gold-standard FSC curve for C22:0-CoA-bound ABCD1 map generated using cryoSPARC 3.1. **d,** Euler angle distribution of the classified particles used for the final 3D refinement of the overall map. **e,** Cryo-EM densities of representative segments of the structure of C22:0-CoA-bound ABCD1. The structure was reconstructed with C2 symmetry, and only one subunit presented. Contour levels are set at 5σ for TM1-6, C22:0-CoA, and at 4σ for CHS. The contour level for the NBDs is set at 3.5σ.

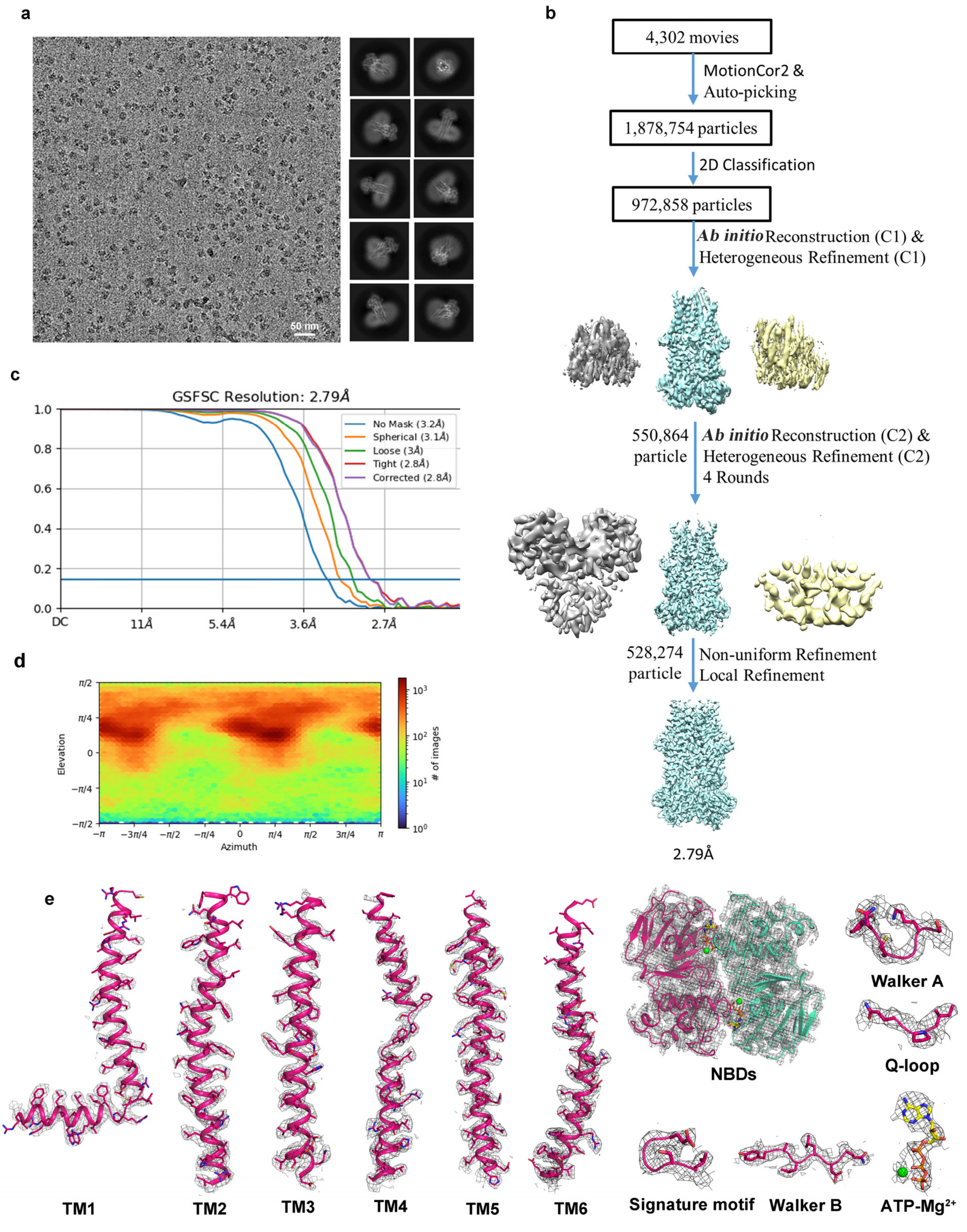
a
50 nm
b
4,302 movies
MotionCor2 &
Auto-picking
1,878,754 particles
2D Classification
972,858 particles
Ab initio Reconstruction (C1) &
Heterogeneous Refinement (C1)
550,864
particle
Ab initio Reconstruction (C2) &
Heterogeneous Refinement (C2)
4 Rounds
528,274
particle
Non-uniform Refinement
Local Refinement
2.79Å
c
GSFSC Resolution: 2.79Å
No Mask (3.2Å)
Spherical (3.1Å)
Loose (3Å)
Tight (2.8Å)
Corrected (2.8Å)
1.0
0.8
0.6
0.4
0.2
0.0
DC
11Å
5.4Å
3.6Å
2.7Å
d
Elevation
Azimuth
# of images
e
TM1
TM2
TM3
TM4
TM5
TM6
NBDs
Walker A
Q-loop
Signature motif
Walker B
ATP-Mg2+

Supplementary Fig. 4 Data processing and model building of ATP-bound ABCD1. a, Representative cryo-EM micrographs and 2D averages. Bar: 50 nm. The micrograph is a representative of 4,302 cryo-EM images. **b,** Flowchart for cryo-EM data processing. **c,** Gold-standard FSC curve for ATP-bound ABCD1 map generated using cryoSPARC 3.1. **d,** Euler angle distribution of the classified particles used for the final 3D refinement of the overall map. **e,** Cryo-EM maps for representative segments of ATP-bound ABCD1. The structure was imposed with C2 symmetry, so only segments in one subunit are presented. Contour levels are set at 5σ for TM1-6, NBDs, ATP-Mg^{2+} molecules and the consensus motif in NBDs including Walker A, Q-loop, signature motif, and Walker B.

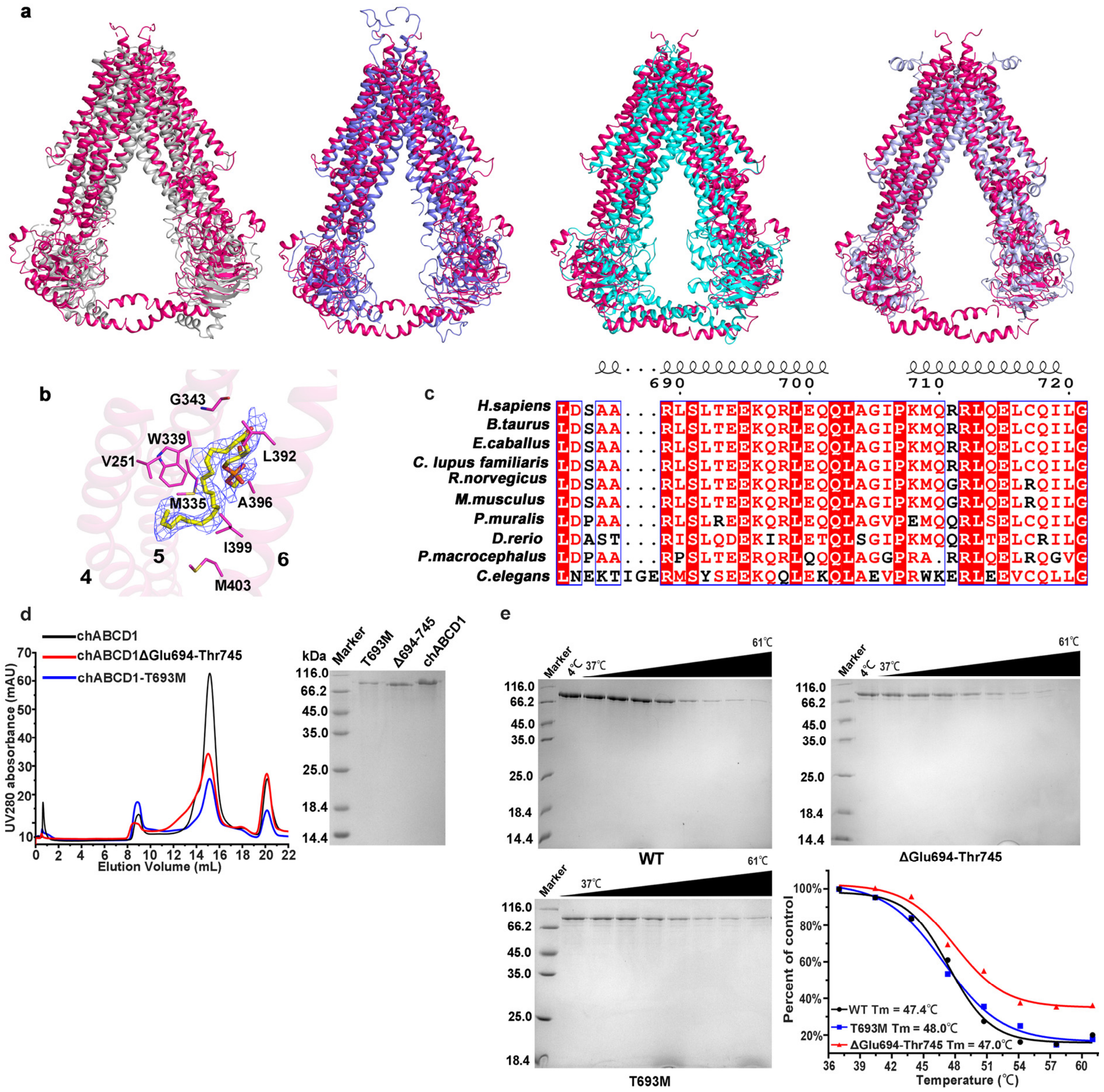

Supplementary Fig. 5 Structural and biochemical characterizations of the apo-form ABCD1. **a,** Structural alignment of ABCD1 and the other four type-IV ABC transporters, yielding an RMSD of 9.6, 13.7 10.7 and 26 Å, over 829, 858, 871 and 797 Cα, respectively. Human ABCD1, ABCB6 (PDB ID: 7EKM), ABCB11 (PDB ID: 6LR0), *Saccharomyces cerevisiae* Atm1 (PDB ID: 7SPL) and *Campylobacter jejuni* PglK (PDB ID: 5c76) are colored in rose, gray, marine, cyan and lightblue, respectively. **b,** Density map for a phosphatidyl ethanolamine (PE) molecule**.** A PE

molecule (contour level at 5σ) was inserted into the hydrophobic cleft formed by TM5 and TM6. **c,** Multiple-sequence alignment for the C-terminal helix of ABCD1 and homologs. **d**, The size exclusion chromatography profiles of chABCD1 and mutants of ΔGlu694-Thr745 and T693M using a Superose 6 Increase 10/300 SEC column. The peak fractions around 15mL were applied to SDS-PAGE and visualized by Coomassie-blue staining. The chromatogram and SDS-PAGE are representatives of > 3 independent experiments that showed similar results **e,** The thermal stability assays of chABCD1 and mutants of ΔGlu694-Thr745 and T693M. One time thermal stability assay of chABCD1 and mutants of ΔGlu694-Thr745 and T693M was performed in this project (n=1). Source data are provided as a Source Data file.

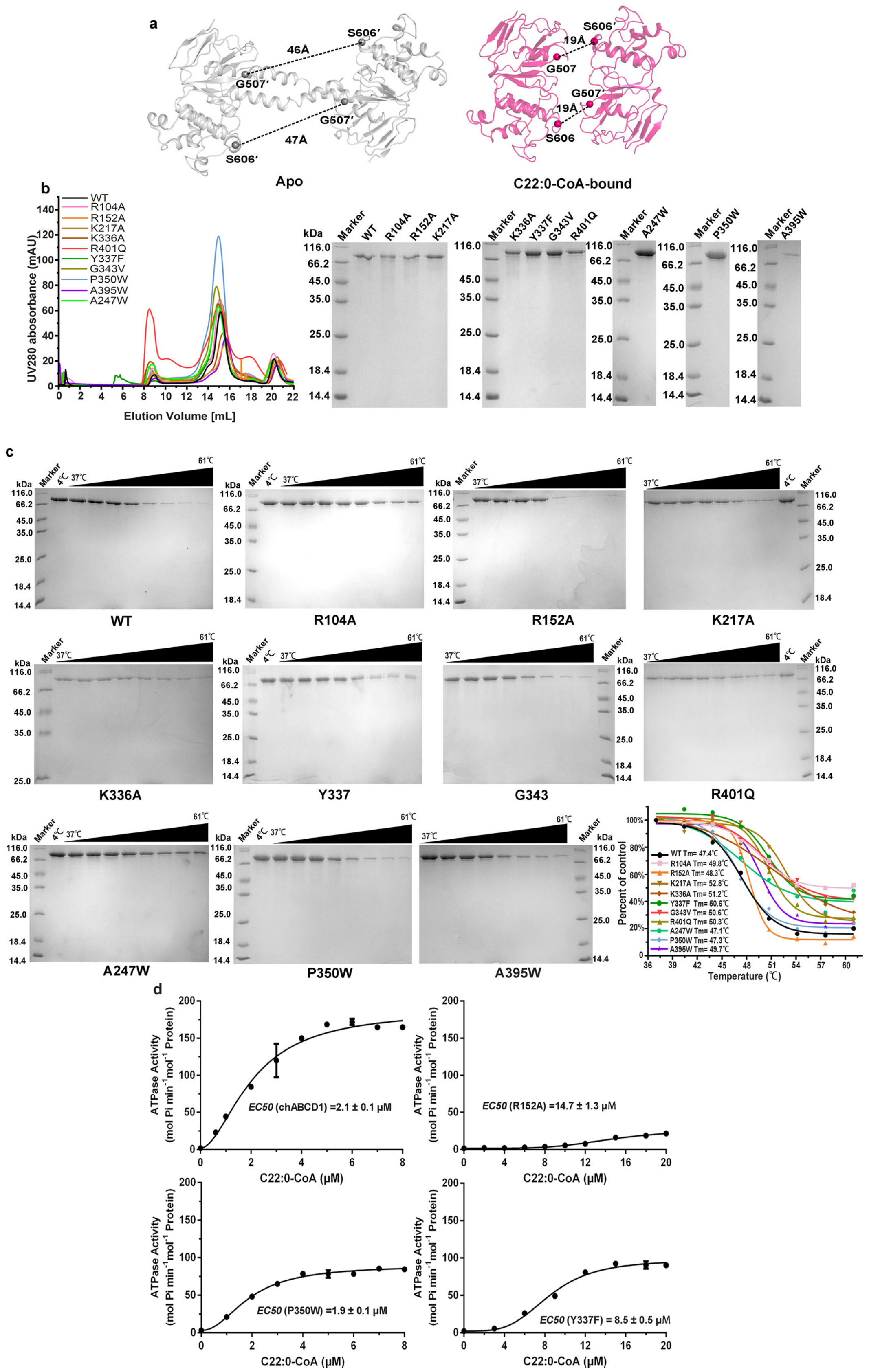

a
Apo
C22:0-CoA-bound
b
Elution Volume [mL]
UV280 abosorbance (mAU)
c
WT
R104A
R152A
K217A
K336A
Y337
G343
R401Q
A247W
P350W
A395W
Temperature (℃)
Percent of control
d
EC50 (chABCD1) =2.1 ± 0.1 μM
EC50 (R152A) =14.7 ± 1.3 μM
EC50 (P350W) =1.9 ± 0.1 μM
EC50 (Y337F) = 8.5 ± 0.5 μM
C22:0-CoA (μM)

77

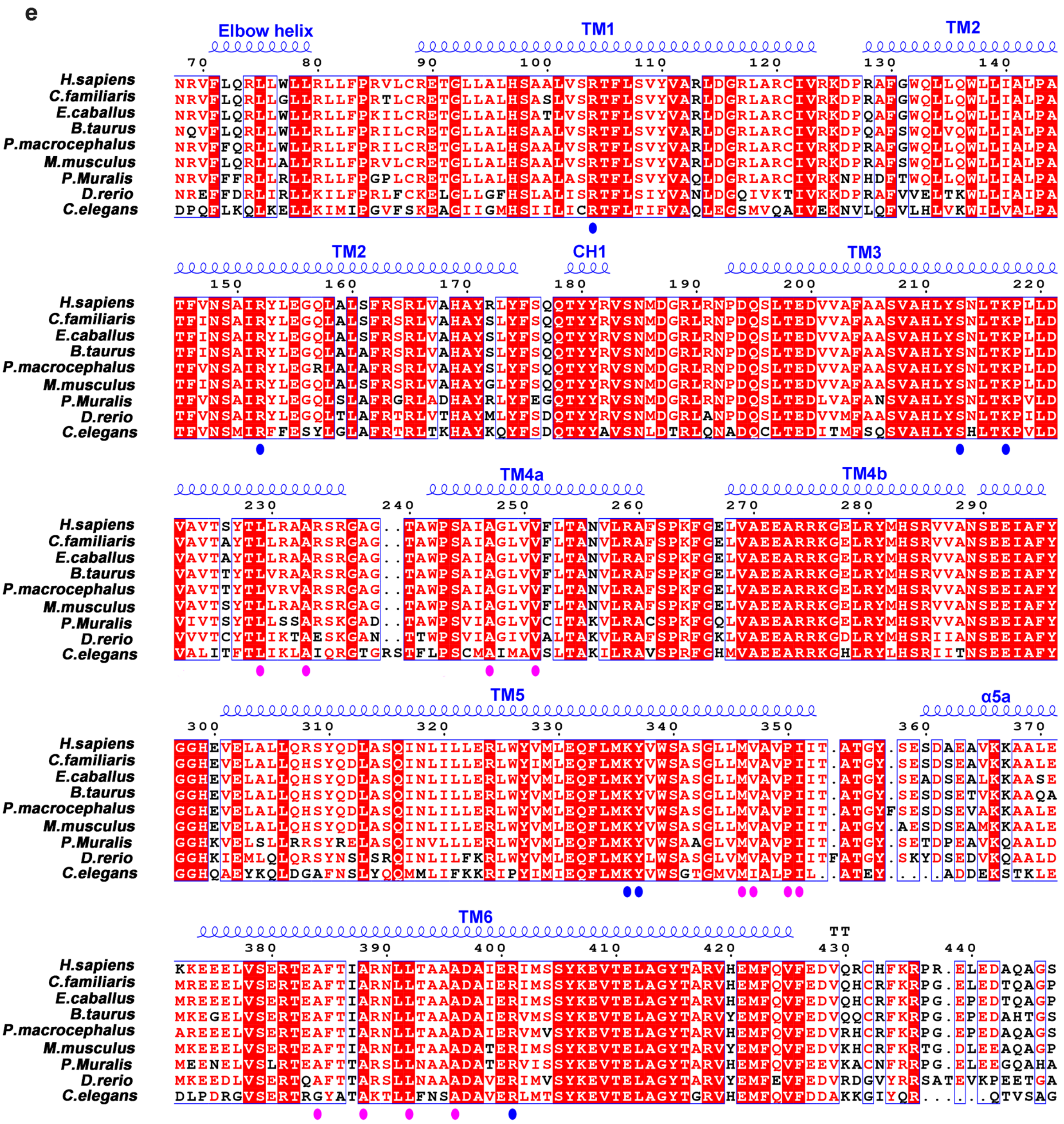

Supplementary Fig. 6 Conformational changes of ABCD1 upon substrate binding. a, The NBDs in apo-form and C22:0-CoA-bound ABCD1, viewed from the peroxisome matrix. The Cα distances between the Walker A and the signature motif are indicated. **b**, The size exclusion chromatography profiles of chABCD1 and mutants related to substrate binding using a Superose 6 Increase 10/300 GL SEC column. The peak fractions around 15 mL were applied to SDS-PAGE

and visualized by Coomassie-blue staining. The chromatogram and SDS-PAGE are representatives of > 3 independent experiments that showed similar results. **c,** The thermal stability assays of chABCD1 and mutants related to substrate binding. One time thermal stability assays of chABCD1 and mutants was performed in this project (n=1). Source data are provided as a Source Data file. **d,** Substrate concentration-dependent ATPase activity of chABCD1 and mutants R152A, Y337F and P350W in detergent of LMNG+CHS, upon addition of C22:0-CoA. The data points were fitted with a Hill equation, based on which EC_{50} were calculated. All data points represent means of three independent measurements (n=3). Error bars indicate standard deviation. Source data are provided as a Source Data file. **e,** Multiple-sequence alignment of ABCD1 and homologs. Circles below the alignment indicate residues interacting with the CoA portion (blue) or the fatty acyl chain (pink) portion of the substrate.

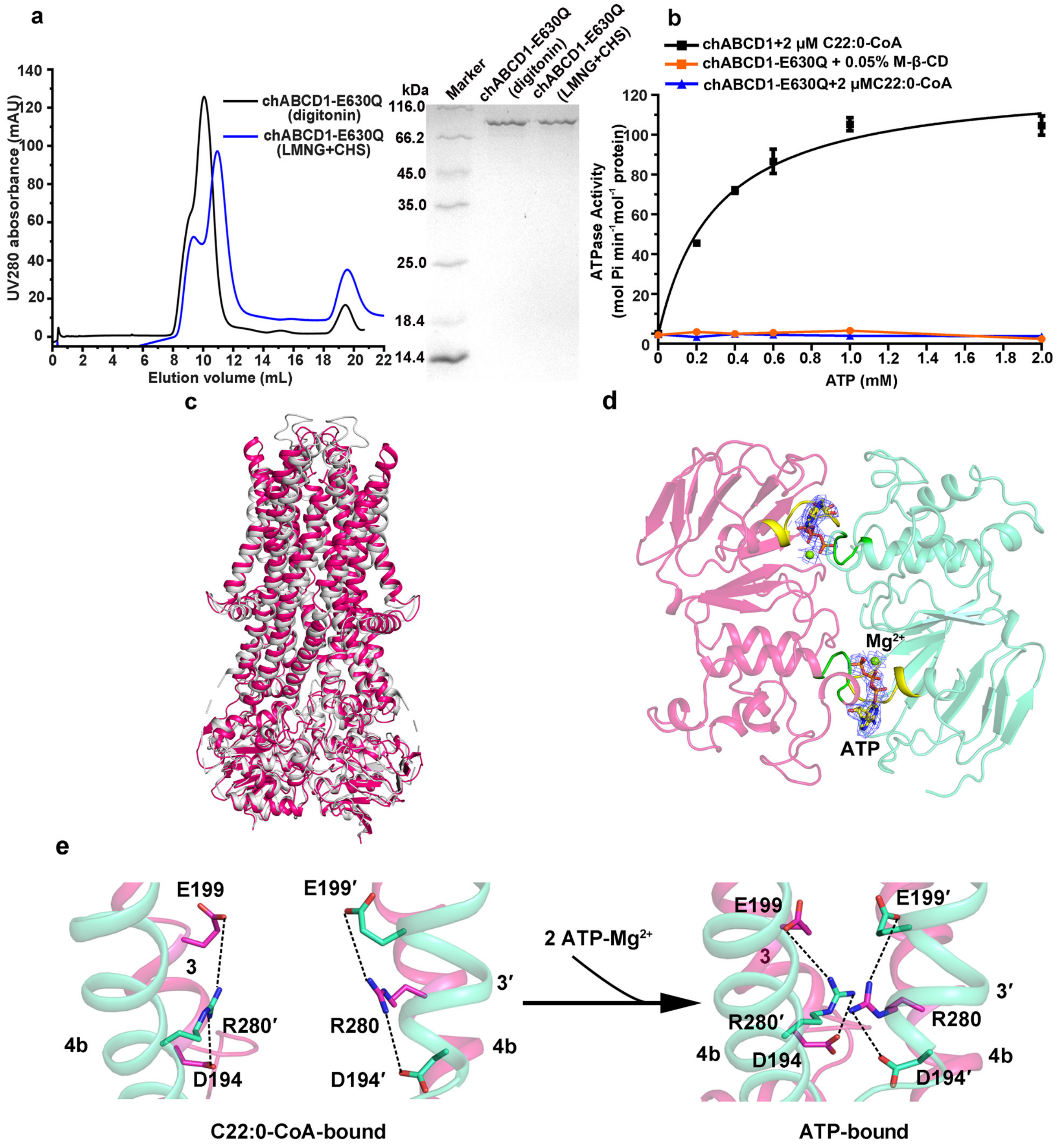

Supplementary Fig. 7 The structural characterization of ATP-bound ABCD1. a, The size exclusion chromatography profiles of chABCD1-E630Q in detergent of digitonin and LMNG+CHS with a Superdex 200 10/300 GL SEC column. The peak fractions were applied to SDS-PAGE and visualized by Coomassie-blue staining. The chromatogram and SDS-PAGE are

representatives of 3 independent experiments that showed similar results. **b,** ATPase activities of chABCD1-E630Q in LMNG+CHS in the presence or absence of C22:0-CoA. 0.05% M-β-CD was also tested as the control group. The data points were fitted with a Michaelis-Menten equation. All data points represent means of three independent measurements (n=3). Error bars indicate standard deviation. Source data are provided as a Source Data file. **c,** Superposition of ATP-bound ABCD1 (rose) and ATP-bound ABCD4 (PDB ID:6jbj, gray). **d,** The density maps for ATP-Mg^{2+} molecules are contoured at 5σ. ATP molecules are shown as yellow sticks; Mg^{2+} ions are displayed as green spheres. Walker A and signature motif are colored in yellow and green, respectively. **e,** The TM3/TM3′ and TM4b/TM4b′ in C22:0-CoA- and ATP-bound ABCD1 are shown in cartoon, respectively. The residues at the cytosolic gate are shown as sticks and salt bridges are shown as dotted lines.

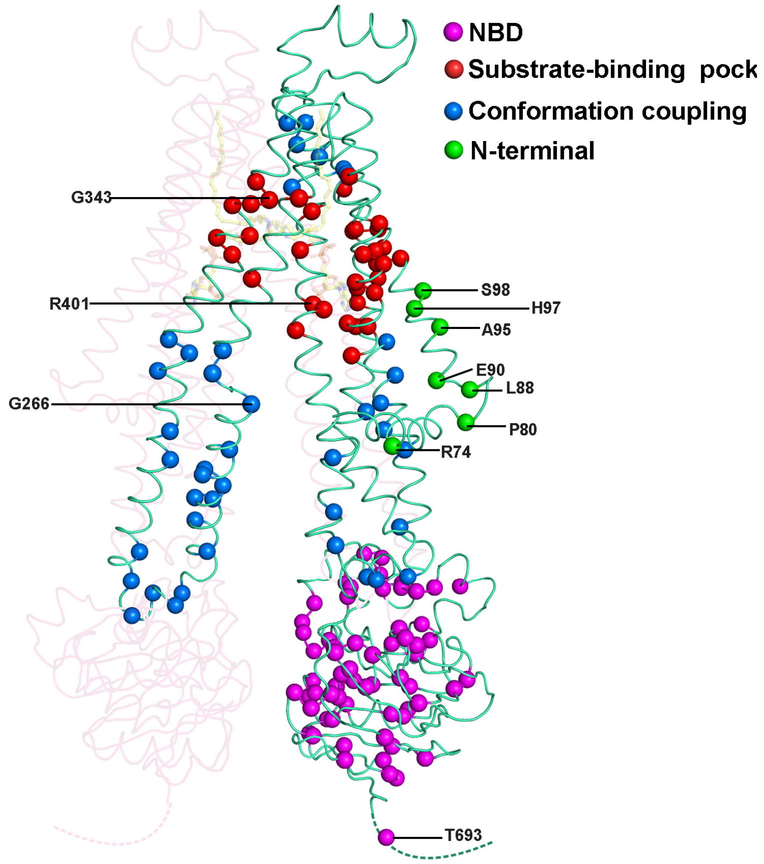

Supplementary Fig. 8 Distribution of X-ALD associated pathogenic variants in ABCD1. The four groups of mutant residues are represented as dots in different colors. The unfolded C-terminus of ABCD1 are shown as dotted line. The pathogenic variants discussed in this manuscript are labelled.

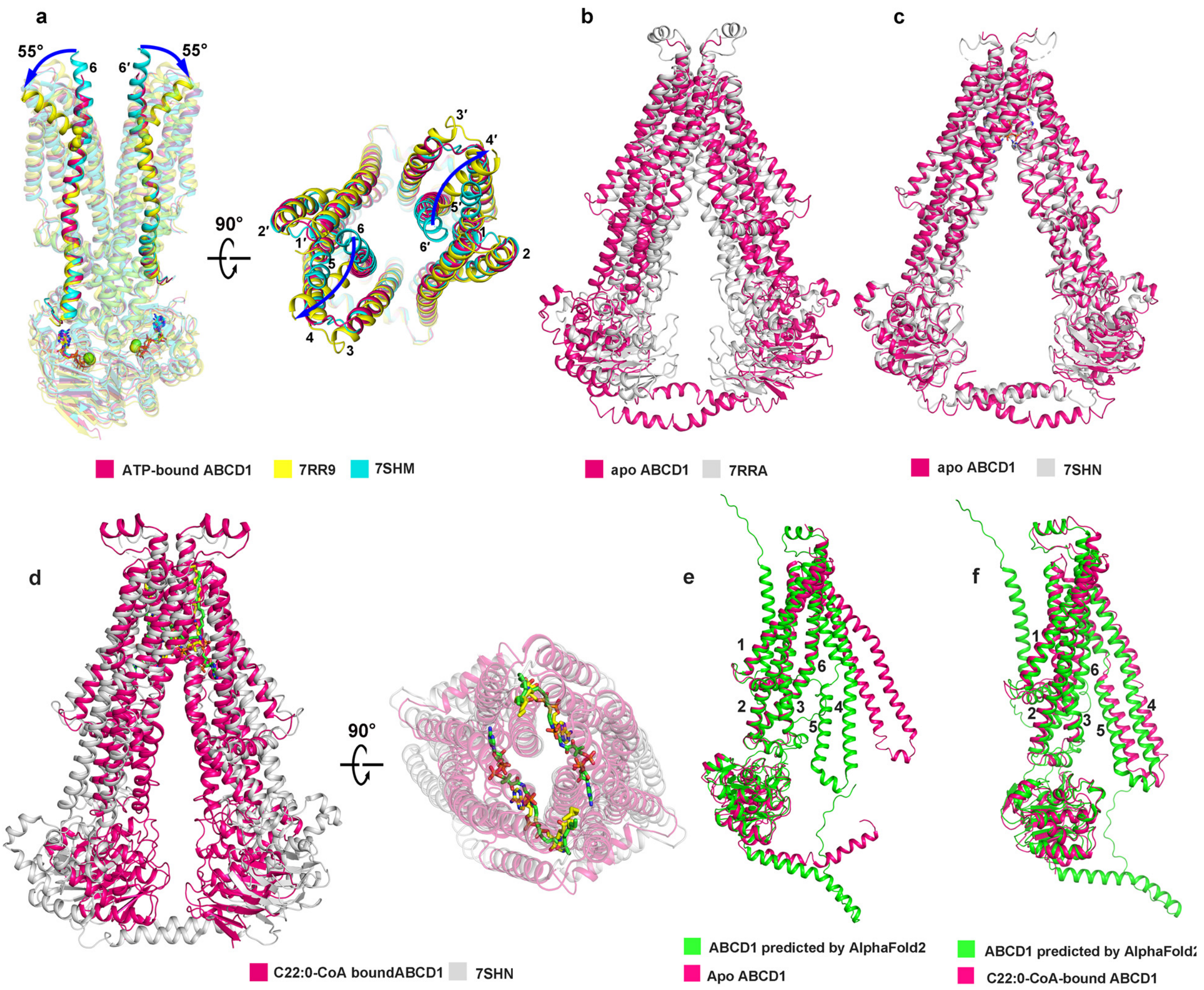

Supplementary Fig. 9 Structural superpositions of our ABCD1 structures against the recently reported ABCD1 structures (labelled with corresponding PDB codes). a, Structural superposition of reported ATP-bound ABCD1 structures. **b** and **c**, Structural superposition of our apo-form ABCD1 structure with the other reported apo-from structures respectively. **d**, Structural superposition of our apo-form structure with the oleyl-CoA-bound structure. The oleyl-CoA and C22:0-CoA are shown as yellow and green sticks, respectively. **e** and **f**, Structural superposition of our apo-form ABCD1 protomers with predicated ABCD1 protomer by AlphaFold2. Protomers form apo-form ABCD1 and C22:0-CoA-bound ABCD1 are superposed with the predicated protomer, respectively.

Supplementary Table 1 Cryo-EM data collection, refinement and validation statistics.

	Apo ABCD1 (EMDB-32152) (PDB 7VWC)	C22:0-CoA-bound ABCD1 (EMDB-32224) (PDB 7VZB)	ATP-bound ABCD1 (EMDB-32171) (PDB 7VX8)
**Data collection and processing**			
Magnification	22,500	22,500	81,000
Voltage (kV)	300	300	300
Electron exposure (e–/Å2)	60	60	54
Defocus range (μm)	-2.0 to -1.5	-2.0 to -1.5	-2.0 to -1.8
Pixel size (Å)	1.07	1.07	1.07
Symmetry imposed	C1	C2	C2
Initial particle images (no.)	4,094,944	6,943,977	1,878,754
Final particle images (no.)	360,311	336,741	550,864
Map resolution (Å)	3.5	3.6	2.8
FSC threshold	0.143	0.143	0.143
Map resolution range (Å)	2.9-4.1	2.9-4.1	2.2-3.0
**Refinement**			
Initial model used (PDB code)	*ab initio*	*ab initio*	6jbj
Model resolution (Å)	3.5	3.6	2.8
FSC threshold	0.143	0.143	0.143
Map sharpening B factor ($Å^2$)	-117.5	-185.8	-116.8
Model composition			
Non-hydrogen atoms	9,854	9,463	9,018
Protein residues	1,223	1,172	1,120
Ligands	Two PE molecules	Two CHS and Two C22:0-CoA molecules	Two ATP molecules and Two Mg^{2+}
B factors ($Å^2$)			
Protein	61.77	89.73	39.77
Ligand	51.01	57.83	28.26
R.m.s. deviations			
Bond lengths (Å)	0.010	0.009	0.009
Bond angles (°)	0.975	0.889	0.992
Validation			
MolProbity score	2.06	2.10	1.77
Clashscore	10.52	14.43	7.41
Poor rotamers (%)	0.38	0.00	1.06
Ramachandran plot			
Favored (%)	91.13	91.91	95.13
Allowed (%)	8.87	8.09	4.87
Disallowed (%)	0.00	0.00	0.00

Supplementary Table 2 The X-ALD associated pathogenic variants in ABCD1. Four groups of X-ALD associated pathogenic variants in ABCD1 are listed, which are displayed in Supplementary Fig. 8. The residues in the NBD, substrate-binding pocket, conformation coupling and N-terminal are colored in magenta, red, blue and green, respectively, the same color as those in the Supplementary Fig. 8.

P484D	S633R	R113C, R113P
L493P	V635M	L114P
M501L	S636I	G116R, G116D, G116E
L503P, L503M	D638Y	A119D
G507S, G507D	E640K	R120P
N509I (Walker A motif, ATP-binding residue)	A646P	L154P
G510S, G510D (Walker A motif, ATP-binding residue)	L654P	L158P
C511Y (Walker A motif, ATP-binding residue)	L655P	L160P
G512S,G512D (Walker A motif, ATP-binding residue)	T658I	R163C, Arg163H, Arg163L, Arg163P
K513Q, K513R (Walker A motif, ATP-binding residue)	R660W, R660Q, R660P	L166P
S514R, S514G, S514I, S514N (Walker A motif, Mg^{2+}-binding residue)	L663Q	Y174D, Y174S
S515F (Walker A motif, Mg^{2+}-binding residue)	H667D, H667L	Q178E
L516P	T668P, T668I	Y181C
R518G, R518Y,R518Q (ATP-binding residue)	H669Y, H669R	R182P
L520P	W679R,	R189W
G522R	T693M	D194N, D194H
L523F	S103R	E199K
P525S	R104C, R104H	D200N
G529S, G529D	T105P, T105I,	A205E
K533E, K533N	T107P	S262W
P534S, P534L, P534R	S108L, S108W	P263L
F540	W137S	G266R, G266E
P543S, P543L	A141T	A273E
Q544K,Q544R (Q-loop, Mg^{2+} and ATP-binding residue)	P143A, P143S, P143H, P143L	R275W
Y547C	V147G	K276E
M548V	N148D, N148Y, N148S	G277R, G277W
S552P	S149N, S149R	L279P
R554H	R152S, R152P	R280C, R280L
Q556R	L211P	H283Y, H283D, H283R
Y559C	S213C	R285G, R285P
P560L, P560R	N214D	E291K, E291G, E291D
D561V,	K217E	E292K
M566K, M566K, M556T	P218S	A294T,
L576P	L220P	Y296C
R591Y, R591L, R591P, R591Q (ATP-binding residue)	D221G	G298S, G298D
G594R	V224G	E302K, E302Q,
A597G	L229P	L313P
L605Q	A245D	Q316P
S606P, S606A, S606L (Signature motif, ATP-binding residue)	T254A,254P, T254M	L322P
G607D (Signature motif, ATP-binding residue)	K336M, K336N	R324C
G608S (Signature motif, ATP-binding residue)	Y337H	L325P
E609K, E609G (Signature motif, ATP-binding residue)	W339R, W339S	T382P
A616T, A616V	A341D	R389G
R617S, R617D, R617G, R617S, R617G, R617C, R617H	S342P	R74W
Y620H, Y620C	G343S, G343V	P84L
A626T	L344P	C88W
L628P	L392V	E90K
D629N, D629H	A396T	A95D
E630K (Walker B motif, ATP-binding residue)	R401W, R401G, R401Q	H97P, H97L
C631R,C631Y,C630W	I402N	S98P, S98L, S98W
T632P, T632I	S404P	
