# Supplementary material for: Structural basis of substrate recognition and translocation by human very long-chain fatty acid transporter ABCD1
Source: Nat Commun. 2022 Jun 8;13:3299. doi: 10.1038/s41467-022-30974-5 (PMC9177597; doi:10.1038/s41467-022-30974-5)
Supplement: Supplementary file 3 — Reporting Summary [file 41467_2022_30974_MOESM3_ESM.pdf]

nature portfolio

Corresponding author(s): Cong-Zhao Zhou, Wen-Tao Hou, Yuxing Chen

Last updated by author(s): May 12, 2022

Reporting Summary

Nature Portfolio wishes to improve the reproducibility of the work that we publish. This form provides structure for consistency and transparency in reporting. For further information on Nature Portfolio policies, see our Editorial Policies and the Editorial Policy Checklist.

Statistics

For all statistical analyses, confirm that the following items are present in the figure legend, table legend, main text, or Methods section.

n/a	Confirmed	
☐	☒	The exact sample size (n) for each experimental group/condition, given as a discrete number and unit of measurement
☐	☒	A statement on whether measurements were taken from distinct samples or whether the same sample was measured repeatedly
☐	☒	The statistical test(s) used AND whether they are one- or two-sided<br>*Only common tests should be described solely by name; describe more complex techniques in the Methods section.*
☒	☐	A description of all covariates tested
☒	☐	A description of any assumptions or corrections, such as tests of normality and adjustment for multiple comparisons
☐	☒	A full description of the statistical parameters including central tendency (e.g. means) or other basic estimates (e.g. regression coefficient) AND variation (e.g. standard deviation) or associated estimates of uncertainty (e.g. confidence intervals)
☐	☒	For null hypothesis testing, the test statistic (e.g. F, t, r) with confidence intervals, effect sizes, degrees of freedom and P value noted<br>*Give P values as exact values whenever suitable.*
☒	☐	For Bayesian analysis, information on the choice of priors and Markov chain Monte Carlo settings
☒	☐	For hierarchical and complex designs, identification of the appropriate level for tests and full reporting of outcomes
☒	☐	Estimates of effect sizes (e.g. Cohen's d, Pearson's r), indicating how they were calculated

Our web collection on statistics for biologists contains articles on many of the points above.

Software and code

Policy information about availability of computer code

Data collection	cryo-EM images of ABCD1 and substrate-bound ABCD1 were collected using SerialEM software, and Cryo-EM images of ATP-bound ABCD1 were collected with EPU 2 software.
Data analysis	Cryo-EM image analyses were performed using standard software: MotionCor2, CTFFIND (ver 4), RELION (ver 3.1) and cryoSPARC (ver 3.1) . Atomic model building is done with COOT (Ver 0.8.9.2), followed by iterative refinement with Phenix (Ver 1.18.2). All structures were validated by PHENIX (Ver 1.18.2) and MolProbity (Ver 4.02). ChimeraX (Ver 1.2.5) and Pymol (Ver 2.5.2) were used for preparing the structural figures . Protein sequences were aligned using Multalin (http://multalin.toulouse.inra.fr/multalin/) and the sequence-alignment figures were generated by ESPript3 server (https://espript.ibcp.fr/). Nonlinear curve fitting and One-way ANOVA were performed with Origin 2021b (Academic). Integrated optical density were measured by ImageJ 1.38X.

For manuscripts utilizing custom algorithms or software that are central to the research but not yet described in published literature, software must be made available to editors and reviewers. We strongly encourage code deposition in a community repository (e.g. GitHub). See the Nature Portfolio guidelines for submitting code & software for further information.

Data

Policy information about availability of data

All manuscripts must include a data availability statement. This statement should provide the following information, where applicable:

- Accession codes, unique identifiers, or web links for publicly available datasets
- A description of any restrictions on data availability
- For clinical datasets or third party data, please ensure that the statement adheres to our policy

The cryo-EM density maps of three structures have been deposited at the Electron Microscopy Data Bank under accession codes: EMD-32152 [https://

www.ebi.ac.uk/emdb/entry/EMD-32152] for apo-form ABCD1, EMD32224[https://www.ebi.ac.uk/emdb/entry/EMD-32224] for C22:0-CoA-bound ABCD1 and EMD-32171[https://www.ebi.ac.uk/emdb/entry/EMD-32171] for ATP-bound ABCD1 and coordinates have been deposited at PDB under accession codes: 7VWC [https://doi.org/10.2210/pdb7VWC/pdb] for apo-form ABCD1, 7VZB[https://doi.org/10.2210/pdb7VZB/pdb] for C22:0-CoA-bound ABCD1 and 7VX8 [https://doi.org/10.2210/pdb7VX8/pdb] for ATP-bound ABCD1. Source data are provided with this paper for Figs. 1a, 1b, 2c, 3g, and supplementary Figs. 1b, 1e, 5e, 6c, 6d, 7b. Source data are provided with this paper.

Field-specific reporting

Please select the one below that is the best fit for your research. If you are not sure, read the appropriate sections before making your selection.

☒ Life sciences ☐ Behavioural & social sciences ☐ Ecological, evolutionary & environmental sciences

For a reference copy of the document with all sections, see nature.com/documents/nr-reporting-summary-flat.pdf

Life sciences study design

All studies must disclose on these points even when the disclosure is negative.

Sample size	No sample-size calculation was performed and the sample size were chosen based on related literature review and the number of independent experiments required for strong inference of meaningful conclusions.
Data exclusions	No data excluded.
Replication	Three replicates were performed in the activity assays (Fig 1a and 1b, Fig 2c, Fig 3g and Supplementary Fig. 1e,6d,7b). All replicates were successful.
Randomization	The protein samples for the biochemical assays were randomly allocated into experimental groups.
Blinding	Blinding was not applicable.

Reporting for specific materials, systems and methods

We require information from authors about some types of materials, experimental systems and methods used in many studies. Here, indicate whether each material, system or method listed is relevant to your study. If you are not sure if a list item applies to your research, read the appropriate section before selecting a response.

Materials & experimental systems

n/a	Involved in the study	
☐	☒	Antibodies
☐	☒	Eukaryotic cell lines
☒	☐	Palaeontology and archaeology
☒	☐	Animals and other organisms
☒	☐	Human research participants
☒	☐	Clinical data
☒	☐	Dual use research of concern

Methods

n/a	Involved in the study	
☒	☐	ChIP-seq
☒	☐	Flow cytometry
☒	☐	MRI-based neuroimaging

Antibodies

Antibodies used	FLAG tag mouse monoclonal antibody (Proteintech, Cat#66008-3-Ig, lot:10011066) ;<br>β-actin mouse monoclonal antibody (protintech, Cat#66009-1-Ig, lot: 10011066) .<br>peroxidase-conjugated Affinipure Goat Anti-Mouse IgG(H+L) (Proteintech, Cat#SA00001-1, lot:20000242).
Validation	all the antibodies used in this project have been characterized and authenticated by the vendors (Proteintech,USA).<br>FLAG tag mouse monoclonal antibody (66008-3-Ig) targets DYKDDDDK tag in WB, RIP, IP, IHC, IF, CoIP, ELISA applications and shows reactivity with recombinant protein samples.<br>The specificity of β-actin mouse monoclonal antibody (Cat#66009-1-Ig) was validated in test application such as FC, IF, IHC, IP, WB, ELISA and shows positive activities for samples from human, mouse, rat, hamster, zebrafish, monkey and dog.

Eukaryotic cell lines

Policy information about cell lines

Cell line source(s)	HEK 293F cell line used to express the protein were purchased from Thermo Fisher Scientific (FreeStyle 293-F, R79007).

Authentication	No further authentication was performed for commercially available cell lines.
Mycoplasma contamination	The cell line were not tested for Mycoplasma contamination. All cell lines exhibited normal growth pattern.
Commonly misidentified lines (See ICLAC register)	No such cell lines were used in this study.
